# Early Treatment with Fumagillin, an Inhibitor of Methionine Aminopeptidase-2, Prevents Pulmonary Hypertension in Monocrotaline-Injured Rats

**DOI:** 10.1371/journal.pone.0035388

**Published:** 2012-04-11

**Authors:** Daniel J. Kass, Eileen Rattigan, Rehan Kahloon, Katrina Loh, Liyang Yu, Asaf Savir, Mark Markowski, Anjali Saqi, Revathi Rajkumar, Ferhaan Ahmad, Hunter C. Champion

**Affiliations:** 1 Division of Pulmonary, Allergy, and Critical Care Medicine, Department of Medicine, University of Pittsburgh School of Medicine and the Dorothy P. and Richard P. Simmons Center for Interstitial Lung Disease, Pittsburgh, Pennsylvania, United States of America; 2 Department of Cardiology, Geisinger Health System, Wilkes-Barre, Pennsylvania, United States of America; 3 Division of Pulmonary, Allergy, and Critical Care Medicine, Department of Medicine, Columbia University College of Physicians and Surgeons, New York, New York, United States of America; 4 Department of Pathology, Columbia University College of Physicians and Surgeons, New York, New York, United States of America; 5 Department of Medicine, Cardiovascular Institute, University of Pittsburgh School of Medicine, Pittsburgh, Pennsylvania, United States of America; 6 Vascular Medicine Institute, University of Pittsburgh School of Medicine, Pittsburgh, Pennsylvania, United States of America; McMaster University, Canada

## Abstract

Pulmonary Hypertension (PH) is a pathophysiologic condition characterized by hypoxemia and right ventricular strain. Proliferation of fibroblasts, smooth muscle cells, and endothelial cells is central to the pathology of PH in animal models and in humans. Methionine aminopeptidase-2 (MetAP2) regulates proliferation in a variety of cell types including endothelial cells, smooth muscle cells, and fibroblasts. MetAP2 is inhibited irreversibly by the angiogenesis inhibitor fumagillin. We have previously found that inhibition of MetAP2 with fumagillin in bleomycin-injured mice decreased pulmonary fibrosis by selectively decreasing the proliferation of lung myofibroblasts. In this study, we investigated the role of fumagillin as a potential therapy in experimental PH. *In vivo*, treatment of rats with fumagillin early after monocrotaline injury prevented PH and right ventricular remodeling by decreasing the thickness of the medial layer of the pulmonary arteries. Treatment with fumagillin beginning two weeks after monocrotaline injury did not prevent PH but was associated with decreased right ventricular mass and decreased cardiomyocyte hypertrophy, suggesting a direct effect of fumagillin on right ventricular remodeling. Incubation of rat pulmonary artery smooth muscle cells (RPASMC) with fumagillin and MetAP2-targeting siRNA inhibited proliferation of RPASMC *in vitro*. Platelet-derived growth factor, a growth factor that is important in the pathogenesis of PH and stimulates proliferation of fibroblasts and smooth muscle cells, strongly increased expression of MetP2. By immunohistochemistry, we found that MetAP2 was expressed in the lesions of human pulmonary arterial hypertension. We propose that fumagillin may be an effective adjunctive therapy for treating PH in patients.

## Introduction

Pulmonary Hypertension (PH) is a pathophysiologic condition defined by a mean pulmonary arterial pressure >25 mmHg at rest [1] and is characterized by hypoxemia, exercise limitation, and in many cases, death [2]. PH occurs in a variety of clinical situations and is associated with a broad spectrum of histological patterns and abnormalities [3]. While idiopathic pulmonary arterial hypertension (IPAH) is rare (15 per one million [4]), PH complicates and worsens the prognosis of many systemic diseases including chronic obstructive pulmonary disease, idiopathic pulmonary fibrosis (IPF), human immunodeficiency virus infection, systemic sclerosis (SSc), and liver cirrhosis (reviewed in [5]). A shared pathologic feature of PH and diseases such as IPF or SSc is the expansion of the mesenchymal cell compartment in the lung. The lesions of PH are characterized to various degrees, depending on the particular etiology, by the accumulation of endothelial cells, smooth muscle cells, and fibroblasts in the pulmonary vasculature (reviewed in [6]). Altered rates of proliferation and apoptosis in mesenchymal cells lead to thickened and narrowed vessels resulting ultimately, in increased pulmonary arterial pressures and right ventricular strain [6]. In human PH (reviewed in [7]) and in experimental models of PH [3], [8], [9], smooth muscle and endothelial cells actively proliferate, leading to occlusion of the pulmonary arteries and the stigmata of PH. Therapies that prevent the accumulation of these cells in the pulmonary arterial bed would be predicted to impact positively both quality-of-life and survival in PH. The currently available vasodilatory therapies for IPAH do enhance survival [10], [11] but do not significantly address the root pathological lesions of PH: the accumulation of fibroblasts, smooth muscle and endothelial cells.

Methionine aminopeptidase-2 (MetAP2) is a metalloprotease that functions in the regulation of fibroblast [12], smooth muscle [13], and endothelial cell [14] proliferation. The enzymatic function of MetAP2 is to cleave the N-terminal methionine off polypeptides, a process that is necessary for many downstream, post-translational modifications of certain proteins [15], [16]. Fumagillin is a fungal metabolite [17] that irreversibly inactivates the enzymatic activity of MetAP2 [18], [19]. Fumagillin and its analogues are potent inhibitors of proliferation and have advanced into clinical trials as angiogenesis inhibitors in multiple cancers. There is accumulating evidence that fumagillin may be an effective therapy in animal models of fibrosis. We have previously found that fumagillin decreased bleomycin-induced pulmonary fibrosis in mice by selective inhibition of myofibroblast replication [12]. In addition, the fumagillin analogue TNP-470 has been found to inhibit both liver fibrosis [20] and peritoneal fibrosis [21] in animal models. The role of fumagillin in PH has not previously been explored. We hypothesized that inhibition of MetAP2 with fumagillin would prevent the accumulation of smooth muscle cells in the pulmonary vasculature that leads to PH.

## Materials and Methods

This protocol was approved by the Institutional Animal Care and Use Committees and the Institutional Review Boards of both Columbia University and the University of Pittsburgh. At Columbia University the relevant protocols are: IRB-AAAE4847, Expression of MetAP2 in Pulmonary Hypertension and IACUC-AAAB0235, Modification of Smooth Muscle Cell Proliferation with Fumagillin. At the University of Pittsburgh, the relevant protocols are: IRB-08020021, Genetics, Transcriptional Genomics, and Pharmacogenomics of Pulmonary Hypertension and IACUC-1009954, Modification of Smooth Muscle Cell Proliferation with Fumagillin.

### Reagents

Monocrotaline, fumagillin, bromodeoxyuridine (BrdU), and monoclonal antibodies against α-smooth muscle actin (clone 1A4) were obtained from Sigma (St Louis, MO). SYBR Green dye and Rabbit IgG against MetAP2, and Alexa Fluor 488–conjugated goat anti-mouse IgG were from Invitrogen (Carlsbad, CA). Monoclonal antibody (mAb) against bromodeoxyuridine (BrdU) (clone BMC 9318) was from Roche (Indianapolis, IN). Platelet-Derived Growth Factor (PDGF) was purchased from R&D Systems (Minneapolis, MN). Rabbit polyclonal antibodies against Ki67 and von Willebrand Factor were obtained from Neomarkers (Fremont, CA). A mouse monoclonal antibody directed against rat CD45 (clone OX-1) was obtained from AbdSerotec (Raleigh, NC). A goat antibody directed against CD31 (PECAM-1, cat# M-20) was from Santa Cruz Biotechnology (Santa Cruz, CA). MetAP2- and non-targeting siRNA oligonucleotides were purchased from Dharmacon (Lafayette, CO). Hiperfect and primers specific for human MetAP2 and PPIA were from Qiagen (Valencia, CA).

### Monocrotaline-Induced Pulmonary Hypertension

Male Sprague-Dawley Rats (Harlan, Indianapolis, IA) were purchased at 250 grams of mass. Monocrotaline was dissolved in 0.5N hydrochloric acid, and the pH was slowly adjusted to 7.4 with 0.2N sodium hydroxide. A single subcutaneous injection of monocrotaline (60 mg/kg) or vehicle control was administered on day 0. Fumagillin was dissolved in dimethyl sulfoxide (DMSO) at 10 mg/ml, and then further diluted in RPMI 1640 pH 8.0 to a final concentration of 10% DMSO. Beginning three days (early) or two weeks (late) after monocrotaline injury, fumagillin (0.5 mg/kg) was administered by inhalation. Under isoflurane anesthesia, a gel-loading pipet tip was gently inserted into the nares of the rat, a volume of 125 microliters was slowly introduced, and the volume was inhaled. This dosage of fumagillin and route of administration was chosen, rather than the more commonly used higher doses in rodents (20–100 mg/kg [22], [23], [24], [25]), because intranasal delivery afforded localized pulmonary, rather than systemic delivery. Drug or vehicle control was administered every other day for the duration of the experiment. Animals were weighed weekly. Animals were sacrificed at four or five weeks after monocrotaline injury.

### Measurement of Right Ventricular Systolic Blood Pressure

At four weeks after monocrotaline injury, animals were anesthetized with a combination of ketamine and xylazine. The right internal jugular vein was exposed and a 2-French catheter (Millar Instruments, Houston, TX) was introduced and advanced to the right ventricle. Pressure measurements were transduced using PowerLab 4/30 from ADI Instruments (Colorado Springs, CO).

### Measurement of Hemodynamics and Echocardiography

At five weeks after MCT injury, animals were brought to the phenotyping core of the Vascular Medicine Institute of the University of Pittsburgh. Central venous access was obtained via internal jugular venous cannulation using sterile PE-50 tubing. To obtain *in vivo* hemodynamic measurements, rats were anesthetized with a mixture of ketamine (100 mg/kg) and acepromazine (5 mg/kg). The rats were then ventilated by the insertion of a tracheal cannula attached to a rodent ventilator (Harvard Apparatus, South Natick, MA) set to 90 breaths/min with a tidal volume of 8 ml/kg body weight. The chest was opened with a midline incision and then a four-electrode pressure-volume catheter (Scisence Inc., London, Ontario, Canada) was placed through the right ventricular apex to record chamber volume by admittance and pressure micromanometry as described previously analyzed using customized acquisition software (IOX2; Emka, Falls Church, VA) [26], [27]. Direct Doppler velocities of the pulmonary arterial flow were obtained using a 20 MHz ultrasound probe and analyzed using customized software (Doppler signal processing workstation; DSPW; Indus Instruments, Houston, TX). A silk tie was loosely placed around the inferior vena cava (IVC), and a transient decrease in preload was achieved by a gentle constriction of the tie to obtain ESPVR. A family of pressure-volume loops was generated to allow for the evaluation of the right ventricular end-systolic pressure volume relationship (Ees) and ventricular-arterial coupling (Ees/Ea).

### Measurement of Cardiomyocyte Cross-Sectional Area and Vessel Thickness

Sections of the free wall of the right ventricle were fixed in 10% neutral buffered formalin. Five µm sections of paraffin-embedded tissue were cut and routine hematoxylin and eosin staining was performed. The section was scanned by a blinded reviewer for cells cut in cross section. Cross-sectional area was measured using an Olympus CH2 microscope with a DP25 camera and DP2-BSW software (Tokyo, Japan). Modified Movat's pentachrome staining was performed on paraffin-embedded samples of rat lung sections using a kit from Electron Microscopy Services (Hatfield, PA). The pulmonary vessels <250 µm were identified by morphological features and their proximity to airways. Twenty-five vessels in cross-sectional diameter were assessed for each rat sample by a blinded reader. Medial diameter of the vessel wall was measured by determining the distance between the internal and external elastic laminae, stained black by the Movat stain. The widest diameter of the vessel was used. The percent medial thickness is reported as percentage of the medial diameter to the diameter of the entire vessel. Measurements were made at 400× magnification using an Olympus DP25 microscope camera.

### Rat Pulmonary Artery Smooth Muscle Cell Culture and Immunoblotting

Rat pulmonary artery smooth muscle cells (RPASMC) were provided generously by Dr. Brian Zuckerbraun, University of Pittsburgh. The isolation of RPASMC has been described elsewhere [28]. Cells were cultured in low-glucose DMEM:F-12 (1∶1) supplemented with 4 mM glutamine (Glutamax, from Invitrogen). Cells were used between the fourth and eighth passages. For immunoblotting, cells were plated in 24-well plates. On the next day, cells were cultured in serum-free DMEM:F12 overnight. On the next day, cells were stimulated with PDGF at 10 ng/mL. 24 h after the addition of PDGF, cells were lysed in RIPA buffer (150 mM NaCl, 1% NP-40, 0.25% sodium deoxycholate, 0.1% sodium dodecyl sulfate (SDS), 1 mM EDTA, 5 mM PMSF, 50 µg/ml leupeptin, 50 µg/ml aprotinin, 50 mM Tris-HCl, pH 7.4), and subjected to SDS-polyacrylamide gel electrophoresis and immunoblotting with a rabbit polyclonal antibody against MetAP2 and a goat polyclonal against β-actin as previously described [12]. Quantification of band intensity was performed with ImageJ software (http://rsb.info.nih.gov/ij/).

### Cell Proliferation Assays

RPASMC were washed and incubated in DMEM:F12 without FBS for 24 h to synchronize the cells in G0 [29]. To release RPASMC from G0 arrest, the media were then replaced with DMEM:F12 + 10% FBS supplemented with fumagillin 20 nM dissolved in DMSO or vehicle control. RPASMC were also grown with or without PDGF at 10 ng/mL and incubated for 24, 48, and 72 h. To facilitate counting cells, RPASMC were fixed in 3.7% formaldehyde, permeabilized in 0.2% Triton X-100, and stained with the DNA-sensitive dye SYBR green (Invitrogen). SYBR green fluorescence was quantitated with a plate-reading spectrofluorimeter. We have found and published previously that SYBR green fluorescence intensity correlated with cell counts in a hemacytometer. All data represent fold changes in cell number following background subtraction [29].

For quantification of BrdU incorporation, RPASMC were plated in 24-well plates on glass coverslips. Cells were washed and incubated in DMEM:F12 without FBS for 24 h to synchronize the cells in G0 [29]. The media were then replaced with DMEM:F12+10% FBS supplemented with fumagillin dissolved in DMSO or vehicle control, with or without PDGF at 10 ng/mL and incubated for an additional 48 h. Cells were pulsed with BrdU 10 µM for the final 30 minutes. Cells were fixed in 3.7% formaldehyde and permeabilized in 0.2% Triton X-100. To expose the incorporated BrdU, we incubated the fixed cells with 100 U/ml DNase for 60 min at 37°C. Cells were processed for immunofluorescence using a monoclonal antibody against BrdU followed by Alexa Fluor 488–conjugated goat anti-mouse IgG and counterstaining with DAPI. Cells were visualized with a Nikon Eclipse TE200 microscope (Nikon, Tokyo, Japan) equipped with epifluorescence. At least 15 fields were selected at random, and BrdU+ cells were enumerated and scored as a percentage of all cells present/high-power field (hpf). About 1500 cells were counted per experiment.

### Transfection of siRNA

RPASMC were plated in DMEM/F12 and 10% fetal bovine serum on day 0, and were transfected with 37.5 ng of siRNA oligonucleotides specific for rat MetAP2 or non-targeting controls using Hiperfect (Qiagen) on days 0 and 1. Cells were then incubated in the presence of fumagillin 20 nM or vehicle control for 36 hours. Cells were processed for immunoblotting or determination of BrdU incorporation as noted above.

### Immunostaining of Rat and Human Lung

Human lung tissues from patients with Idiopathic Pulmonary Arterial Hypertension (IPAH, World Health Organization, class I) or normal controls were obtained from the Columbia University Tissue Bank. Normal lung was obtained from histologically normal appearing lung distant from resected lung cancers [29]. All diagnoses were rendered by a dedicated lung pathologist according to standard American Thoracic Society criteria. For immunohistochemistry, sections were deparaffinized with xylene followed by rehydration through graded ethanol series. Antigen retrieval with 10 mM sodium citrate was performed for Ki67, MetAP2, and von Willebrand Factor. For brightfield microscopy, antigens were developed with the Vectastain ABC Universal kit (Vector Labs, Burlingame, CA). Diaminobenzidine (brown) and Vector SG (gray) were used for double-labelling experiments. Sections were counterstained with Harris' hematoxylin, dehydrated through graded alcohol series, and mounted with Permount (Fisher, Fairlawn, NJ). Sections were visualized using an Olympus CHS microscope and images were photographed with an Olympus DP25 camera (Olympus, Tokyo, Japan). Ki67+ cells were counted as the number of positively-staining nuclei per vessel per high power field (hpf). Immunostaining for CD45 was performed on cryosections of lungs from rats. CD45+ cells were quantified as the number of positively-staining cells per hpf. Immunofluorescent staining on human lung was performed on paraffin-embedded tissue with rhodamine- and Cy5-conjugated secondary antibodies (Jackson Immunoresearch, West Grove, PA). Nuclei were counterstained with DAPI. Black and white images were photographed on the TE200 Nikon Eclipse, processed, and pseudocolored using Adobe Photoshop.

### Terminal Deoxynucleotidyl Transferase dUTP Nick End Labeling (TUNEL)

Five µm sections of formalin-fixed, paraffin-embedded lung and heart tissues were cut, deparaffinized, and rehydrated through graded ethanol series. Sections were then prepared with proteinase K pre-treatment. TUNEL staining was then performed employing the ApopTag Peroxidase kit (Millipore, Billerica, MA). TUNEL+ cells were counted as the number of positively-staining nuclei per high power field.

### Quantitative RT-PCR

The preparation of samples of cDNA from lungs of patients with pulmonary arterial hypertension (PAH) or normal controls has been described elsewhere [30]. Quantitative RT-PCR was performed, and data were analyzed on the Applied Biosystems 7500 (Carlsbad, CA). Primers specific for human MetAP2 and the endogenous control PPIA were obtained from Qiagen and used according to the manufacturer's recommendations.

### Statistics

Data were analyzed by one-way ANOVA followed by Bonferroni's *post-hoc* test (unless otherwise stated) using GraphPad Prism 5 (GraphPad Software, La Jolla, CA).

## Results

### Inhibition of MetAP2 by Fumagillin Prevents PH in MCT-Injured Rats

**Figure 1:**
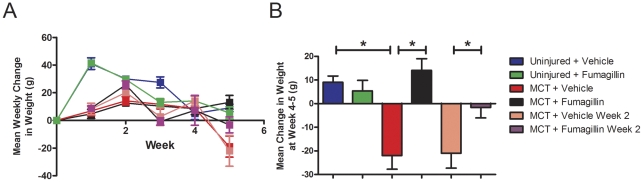
Early Fumagillin Treatment is Associated with Growth in Monocrotaline-Injured Rats. Rats were injected with monocrotaline (MCT) as described in Materials and Methods. Treatment with fumagillin or vehicle controls was initiated on the third day or 14^th^ day after injury and continued every other day for the duration of the experiment. Animals were weighed weekly. At weeks 1–4, all MCT-injured animals gained weight at a similar rate (A). Panel (B) represents the data shown in panel (A) at week 5. MCT-injured rats treated with the vehicle lost nearly 20 g compared with the early fumagillin-treated animals that continued to gain weight at a rate similar to uninjured animals (*P<0.05, MCT+fumagillin v MCT+vehicle, n = 6 per group). In the MCT-injured animals treated with fumagillin starting at week 2, animals exhibited decreased weight loss compared with the MCT-injured animals treated with the vehicle (*P<0.05 MCT+fumagillin week 2 v. MCT + Vehicle, week 2). Data were analyzed by one-way ANOVA followed by the Bonferroni post-hoc test.

We have previously found that delivery of fumagillin to bleomycin-injured mice decreased pulmonary fibrosis by a selective reduction in the number of accumulated fibroblasts in the lung parenchyma. In this study, we sought to expand on our previous findings and determine if treatment of rats with fumagillin would attenuate PH in an animal model where smooth muscle and fibroblast accumulation was a prominent feature. We chose to test the efficacy of fumagillin in the monocrotaline (MCT) model of PH. MCT was injected subcutaneously on day 0, followed by treatment with fumagillin or vehicle control beginning either on day 3 (early treatment), or day 14 (late treatment), after MCT injury. Treatment continued every other day for the duration of the experiment. Animals were weighed weekly as a measure of overall health. All animals gained weight every week for the first four weeks of the experiment (Figure 1A). Between the fourth and fifth weeks, MCT-injured animals, treated with the vehicle all lost nearly 20 grams (Figure 1B). In contrast, MCT-injured rats, treated with fumagillin beginning on day 3 continued to gain weight at a rate similar to uninjured animals. Animals treated with fumagillin beginning 14 days after MCT lost significantly less weight at week 5 than MCT-injured animals treated with the vehicle. These data suggest that treatment with fumagillin initiated at 3 d protected animals from weight loss induced by MCT injury. When fumagillin treatment was initiated at 14 d, we observed attenuated weight loss in response to MCT injury suggesting at least partial protection.

**Figure 2:**
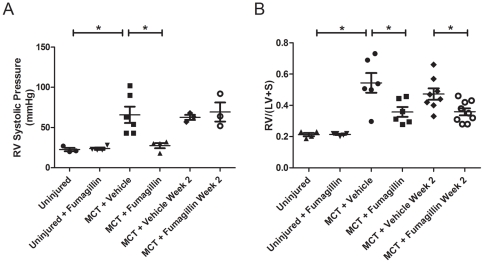
Fumagillin Prevents Pulmonary Hypertension and Right Ventricular Hypertrophy in MCT-Injured Rats. Rats were injected with MCT and treated with fumagillin or vehicle control as described in Materials and Methods. After four weeks, animals were killed, and direct right ventricular pressure was measured (A). MCT injury significantly increased RV systolic BP, which is prevented by early, but not late, treatment with fumagillin (*P<0.05, one-way ANOVA followed by Bonferroni's post hoc test). (B) Measurement of right ventricular mass normalized to left ventricular mass (RV/LV+S). Five weeks after MCT injury, hearts were excised, fixed in formalin, and the masses of the RV and the LV+septum were determined. MCT-injured animals treated with the vehicle exhibited a significant increase in RV mass, which was prevented in both early and late fumagillin-treated animals (*P<0.05, ANOVA followed by Bonferroni's post-hoc test).

Next, we assessed the effects of fumagillin treatment on MCT-injured animals using physiologic and histopathologic measures of PH. On day 28 after injury, right ventricular systolic blood pressure (RVSP) was determined (Figure 2A). RVSP was determined by catheterization of the right internal jugular vein. Average RVSP was less than 25 mmHg in uninjured animals treated with fumagillin or the vehicle control. In MCT-injured, vehicle-treated animals, the average RVSP was 66 mmHg. However, in the MCT-injured animals treated with fumagillin early after injury, the average RVSP was significantly reduced at 28 mmHg. In contrast, animals treated with the vehicle or fumagillin beginning at 14 d after MCT exhibited similarly elevated RVSPs (63 vs. 69 mmHg, respectively). Thus, only early treatment with fumagillin prevented MCT-induced pulmonary hypertension. Treatment of animals with fumagillin beginning at 14 d after MCT injury did not prevent PH.

We next determined the effect of fumagillin on heart size in MCT-injured rats. At five weeks after MCT injury, the hearts and lungs were excised and prepared for gross and histopathologic analysis. We measured the mass of the right ventricle normalized to the mass of the left ventricle and the interventricular septum (Figure 2B). Compared to uninjured controls, the ratio of RV mass to LV mass increased 2.5-fold with MCT injury and treatment with the vehicle. Early treatment with fumagillin attenuated the increase in right ventricular mass induced by MCT injury. Surprisingly, late treatment with fumagillin, beginning 14 d after MCT injury, also attenuated right ventricular remodeling induced by MCT despite RVSPs as high as the vehicle-treated and MCT-injured rats. These data suggest that fumagillin treatment as early as three days and as late as 14 days after MCT injury prevents RV remodeling due to PH.

### Fumagillin Treatment Preserves Right Ventricular Function in MCT-Injured Rats

**Figure 3:**
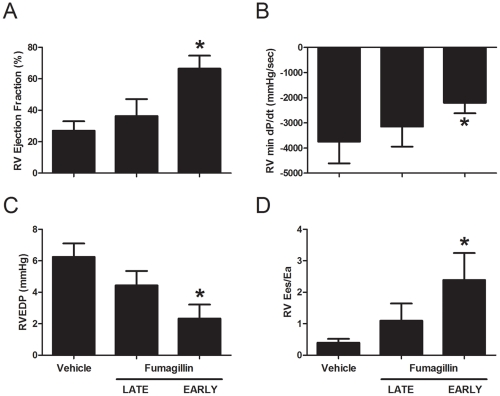
Treatment of monocrotaline-injured rats with fumagillin leads to improved right ventricular function. Rats were injected with MCT and treated with fumagillin or vehicle control beginning at 3 d or 14 d after MCT injury as described in Materials and Methods. At five weeks after MCT injury, animals were prepared for echocardiography and measurement of invasive hemodynamics as described in Materials and Methods: (A) right ventricular ejection fraction, (B) right ventricular minimum dP/dt, (C) right ventricular end-diastolic pressure, and (D) the ratio of right ventricular to pulmonary artery elastance, Ees/Ea. Note stepwise improvement in these hemodynamic parameters as fumagillin treatment is delivered late vs. early (*P<0.05, MCT+early fumagillin v. MCT+vehicle, by t-test n = 4−7 animals per group).

The decreased weight loss exhibited by MCT-injured animals treated with fumagillin late and the decrease in the RV size in these animals in the presence of elevated RVSP suggested to us that fumagillin may offer a direct protective effect on the hearts of animals injured with MCT. We next measured hemodynamics in animals both injured with MCT and treated with fumagillin (Figure 3). Measures of systolic function, including cardiac output and ejection fraction, between uninjured animals treated with the vehicle or fumagillin were identical (data not shown). We found that early treatment with fumagillin significantly increased RV ejection fraction (RVEF) 2.4-fold compared to vehicle-treated, MCT-injured controls (Figure 3A). The mean RVEF was lower in the late fumagillin-treated animals compared to the early treatment, but this difference was not statistically significant. We next measured minimum dP/dt as a measurement of diastolic function in the RV (Figure 3B). We observed a similar trend: early treatment with fumagillin in MCT-injured animals was associated with a statistically significant 1.7-fold improvement in minimum dP/dt as compared to vehicle-treated, MCT-injured controls. Similar to RVEF, min dP/dt was lower in the late fumagillin-treated animals compared to the early treatment, but this difference too was not statistically significant. Early treatment with fumagillin was also associated with a significant reduction in RV end-diastolic pressure (RVEDP) (Figure 3C), a measure of RV failure, in comparison to MCT-injured and vehicle-treated controls. Early treatment with fumagillin also led to a significant increase in the ratio of RV end-systolic volume to stroke volume (Ees/Ea) (Figure 3D), a measure of right ventricular and pulmonary artery coupling [31]. Again, late treatment was associated with measurements that were intermediate between vehicle treatment and early fumagillin treatment. Thus, treatment with fumagillin is associated with improved hemodynamics in MCT-injured animals. The most significant improvements in hemodynamics were observed in animals treated early with fumagillin after MCT injury.

### Fumagillin Inhibits Cardiac Myocyte Hypertrophy in MCT-Induced PH

**Figure 4:**
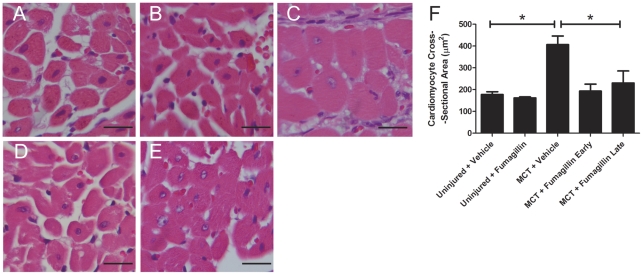
Late treatment with fumagillin inhibits RV cardiomyocyte hypertrophy. Rats were injected with MCT and treated with fumagillin or vehicle control beginning at 14 d after MCT injury as described in Materials and Methods. At five weeks after MCT injury, animals were killed and the free wall of the right ventricles were prepared for histology: (A) Uninjured + vehicle, (B) uninjured + fumagillin, (C) MCT + vehicle, week 2, (D) MCT + fumagillin early, and (E) MCT + fumagillin late (magnification ×400, bar = 20 µm). The area of cardiomyocytes cut in cross section was measured as described in Materials and Methods (F). MCT injury led to a significant increase in cardiomyocyte cross-sectional area, which was attenuated by treatment with fumagillin (*P<0.05, one-way ANOVA, followed by Bonferroni's post-hoc test).

Because we found that late treatment with fumagillin decreased RV mass in the presence of high pulmonary arterial pressures, we next measured the cross-sectional diameter of cardiac myocytes in animals injured with MCT and treated with the vehicle or with late fumagillin (14 d after MCT) (Figure 4). No significant difference in cardiac myocyte diameter was found between uninjured animals treated with the vehicle or with fumagillin. After MCT injury, myocyte diameter in vehicle-treated animals significantly increased 2.3-fold compared to the uninjured control. Late treatment with fumagillin significantly attenuated the increase in myocyte diameter induced by MCT by 76%. Thus fumagillin has a direct effect on the cardiomyocyte hypertrophy in MCT-injured animals.

### Late Fumagillin Does Not Affect Capillary Number in the Right Ventricles of MCT-Injured Rats

**Figure 5:**
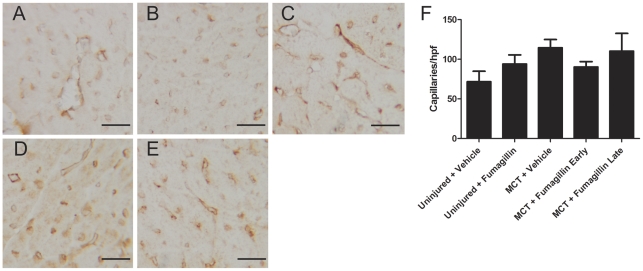
Treatment with fumagillin is not associated with differences in capillary density in MCT-injured animals. Rats were injected with MCT and treated with fumagillin or vehicle control beginning at 14 d after MCT injury as described in Materials and Methods. At five weeks after MCT injury, animals were killed and the free wall of the right ventricles were prepared for immunostaining for PECAM: (A) Uninjured + vehicle, (B) uninjured + fumagillin, (C) MCT + vehicle, week 2, and (D) MCT + fumagillin early, and (E) MCT + fumagillin late (magnification ×400, bar = 20 µm). The number of microvessels was counted per hpf as described in Materials and Methods (F). No significant differences in the number of microvessels were detected in fumagillin-treated animals compared to vehicle controls (data analyzed by one-way ANOVA, followed by Bonferroni's post-hoc test, n = 3–5 animals per group).

To investigate further the effect of fumagillin on the hearts of MCT-injured animals, we performed immunohistochemistry for CD31 (PECAM) to quantify the number of capillaries in the hearts of these animals (Figure 5). We found that vehicle treatment in MCT-injured animals was associated with an increase in the number of capillaries compared to vehicle-treated uninjured controls. Early treatment with fumagillin resulted in a borderline reduction in capillary number, but late treatment with fumagillin did not affect capillary number in the heart in comparison to the MCT-injured, vehicle-treated controls. These data suggest that late treatment of MCT-injured animals with fumagillin does not impair right ventricular neoangiogenesis in response to pulmonary hypertension.

### Fumagillin Decreases Apoptosis in the Right Ventricles of MCT-Injured Rats

**Figure 6:**
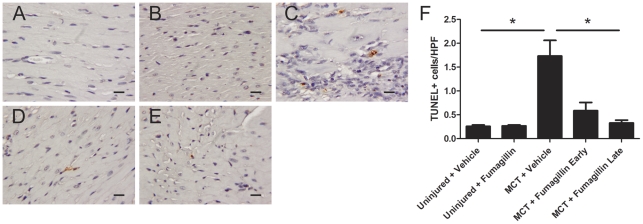
Treatment with fumagillin decreases the number of apoptotic cells in rat hearts. Rats were injected with MCT and treated with fumagillin or vehicle control beginning at 14 d after MCT injury as described in Materials and Methods. At five weeks after MCT injury, animals were killed and the free wall of the right ventricles were prepared for TUNEL staining: (A) Uninjured + vehicle, (B) uninjured + fumagillin, (C) MCT + vehicle, week 2, (D) MCT + fumagillin early, and (E) MCT + fumagillin late (magnification ×400, bar = 20 µm). The number of TUNEL+ cells in the right ventricles were increased by MCT injury but attenuated by fumagillin treatment (F) (*P<0.05, one-way ANOVA, followed by Bonferroni's post-hoc test, n = 3–5 animals per group).

We next examined histologic sections of the right ventricles of MCT-injured animals to determine if fumagillin affected the rate of apoptosis in injured hearts. We performed terminal deoxynucleotidyl transferase dUTP nick end labeling (TUNEL) on rat hearts following MCT injury and treatment at different time points with fumagillin (Figure 6). Following MCT injury, there was a significant increase in the number of apoptotic cells in the vehicle-treated animals that was blocked by either early or late treatment with fumagillin.

### Early Treatment with Fumagillin Decreases Medial Thickness of the Pulmonary Arteries

**Figure 7:**
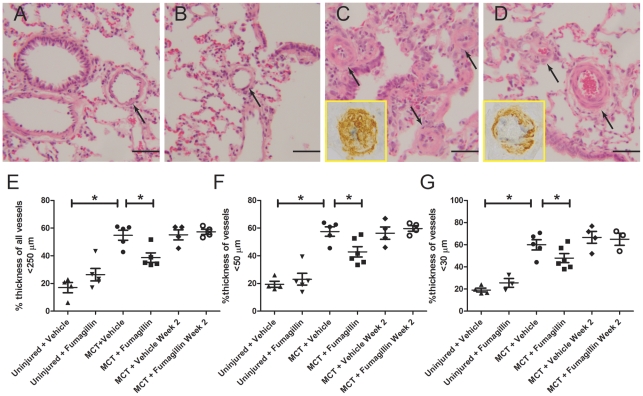
Fumagillin Protects Rats from PA thickening in MCT-injured Rats. Rats were injected with MCT and underwent treatment with fumagillin or vehicle control as described in Materials and Methods. Animals were killed on day 35 after injury, and the lungs were processed for histology. Hematoxylin and eosin staining of lungs from (A) uninjured, vehicle-treated (B) uninjured, fumagillin-treated, (C) MCT-injured, vehicle treated, or (D) MCT-injured, early fumagillin treated. Arrows point to thickened pulmonary arteries (×100 magnification, scale bar = 50 µm). Inset images are 400× magnification and show immunohistochemistry for α-SMA (brown) and vWF (gray) as described in Materials and Methods. Pulmonary artery thickness was measured by Movat staining as described in Materials and Methods (Representative images are shown in Figure S1) (E) All vessels <250 µm, (H) vessels <50 µm, (I) vessels <30 µm. *P<0.05, n = 4–6 animals). Data were analyzed by one-way ANOVA followed by the Bonferroni post-hoc test.

Next, we examined histologic sections of the lung from MCT-injured animals to determine the effects of fumagillin treatment on the pulmonary vasculature. We hypothesized that fumagillin would directly block accumulation of smooth muscle cells in MCT-injured rats. Routine hematoxylin and eosin staining was performed on lung sections from uninjured and MCT-injured animals (Figure 7A–D). MCT injury was associated with severe lung injury including architectural distortion and the prominent accumulation of foamy macrophages. In MCT-injured, vehicle-treated animals, significant thickening of the medial layer was observed in all vessels. Some vessels in the vehicle-treated MCT-injured animals were also occluded. In the MCT-injured, early fumagillin-treated animals, the medial layers were thickened, but all vessels observed remained patent. We also performed double-label immunohistochemistry for α-smooth muscle actin (α-SMA) to mark smooth muscle cells and myofibroblasts, and von Willebrand Factor (vWF) to mark endothelial cells (*inset,* Figure 7C, D). In MCT-injured, vehicle-treated animals, there was significant thickening of the medial layer with endothelial cells appearing bunched, and some vessels exhibited extensive muscularization. In contrast, in early fumagillin-treated animals, vessels again were mildly thickened but widely patent. We measured vessel thickness employing Movat staining in the pulmonary vasculature as described in Materials and Methods (Figure 7E–G). Representative Movat images are shown in Figure S1. In all vessels measuring less than 250 µm and in the vessels measuring less than 50 and 30 µm in cross-sectional diameter, MCT injury and vehicle treatment was associated with significant vessel thickening, which was attenuated by early treatment with fumagillin. In the animals receiving late fumagillin therapy, there was no significant difference in vessel thickness between fumagillin- and vehicle-treated animals. These data show that early fumagillin treatment is associated with decreased thickening of the medial layer of the pulmonary arteries in MCT-injured animals. Because late fumagillin treatment did not decrease thickening of the medial layers of the pulmonary vasculature, these data also suggest that the accumulation of cells in the medial layer in MCT-injured animals is an event that occurs within the first 14 days after MCT injury [32].

### Fumagillin Treatment Decreases the Accumulation of CD45+ Cells after Monocrotaline Injury

**Figure 8:**
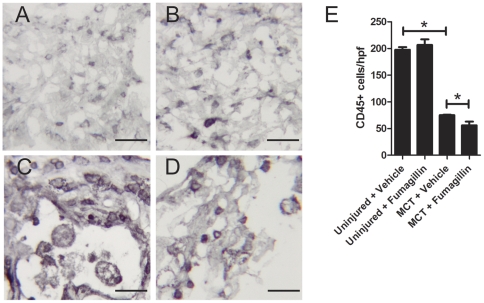
Fumagillin treatment decreases the number of inflammatory cells following monocrotaline injury. Immunohistochemistry was performed for common leukocyte antigen (CD45). CD45+ cells were quantified per high power field (hpf) as described in Materials and Methods. MCT injury was associated with a significant decrease in the number of CD45+ cells in the lung. Early treatment with fumagillin decreased CD45+ cells in the lung by 25% (*P<0.05 by ANOVA, n = 3–4 animals per group).

To understand the effect of fumagillin on inflammation in MCT-injured lungs, we quantified the number of CD45+ cells in the lungs (Figure 8). No significant differences were detected between uninjured animals treated either with the vehicle or fumagillin. After MCT injury, we observed a large reduction in the number of CD45+ cells. Many of the CD45+ cells in the MCT-injured animals appeared to be foamy macrophages. In MCT-injured animals, fumagillin decreased the accumulation of CD45+ cells by 34% compared to vehicle treatment. Thus, fumagillin was associated with reduced inflammation following MCT injury.

### MetAP2 Inhibition by Fumagillin Suppresses Proliferation in Rat PA Smooth Muscle Cells

**Figure 9:**
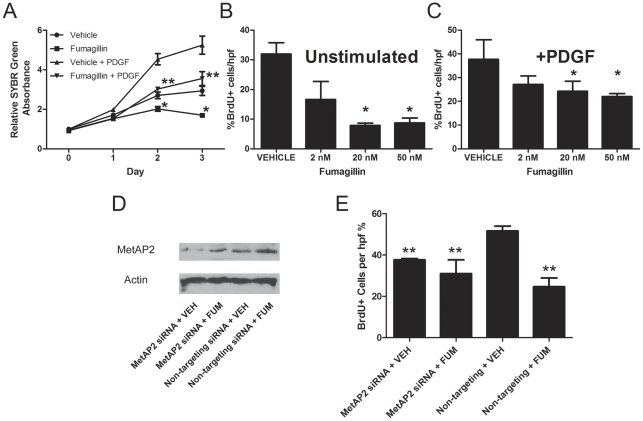
Fumagillin and Silencing of MetAP2 Expression Inhibit Proliferation of RPASMC *in vitro*. (A) Primary RPASMC were serum-starved in basal medium for 24 h to synchronize all cells in G0. Cells were then released from G0 by addition of 10% FBS supplemented with or without PDGF (10 ng/mL) or fumagillin (20 nM). Cells were fixed after 0, 1, 2, and 3 days of culture and then stained with SYBR Green as described in Materials and Methods. Fluorescent intensity, which correlates with relative cell number, was measured by a fluorimetric plate reader. Data represent mean + SEM, n = 4. At days 2 and 3, fumagillin significantly reduced cell number in cells incubated in the absence of PDGF (*P<0.01) or in the presence of PDGF (**P<0.001). (B–C) RPASMC were incubated in the absence of serum followed by addition of serum (B) or serum with PDGF (10 ng/mL) (C) with or without fumagillin at the indicated concentrations. Cells were incubated for an additional 48 h and pulsed with BrdU for the final 30 minutes then fixed. BrdU+ cells were quantified as described in Materials and Methods. Data represent mean + SEM. Data analyzed by ANOVA followed by Tukey's multiple comparison test (*P<0.05, fumagillin v vehicle AND fumagillin + PDGF v. vehicle + PDGF). (D) Immunoblotting for MetAP2 of RPASMC cultured in the presence of 10% serum and exposed to MetAP2-targeting siRNA (lanes 1 and 2) or non-targeting RNA oligonucleotides (lanes 3 and 4). Cells in lanes 1 and 3 were incubated with the vehicle control, and cells in lanes 2 and 4 were incubated with fumagillin 20 nM. Note the increased band intensity of MetAP2 in the fumagillin conditions. (E) Incorporation of BrdU in RPASMC during silencing of MetAP2 gene expression by siRNA. During suppression of MetAP2 gene expression, BrdU incorporation was decreased 27% compared to the non-targeting control (**P<0.05, ANOVA followed by Neuman-Keuls post-hoc test compared to Non-targeting + VEH). In the presence of fumagillin, silencing of MetAP2 did not lead to additional inhibition of proliferation.

Because we found decreased thickening of the pulmonary arteries in MCT-injured rats with fumagillin, we reasoned that fumagillin may inhibit the proliferation of smooth muscle cells directly. We have previously found that inhibition of MetAP2 by fumagillin, or that silenced expression of MetAP2 by short interfering RNA (siRNA), decreased proliferation of rat lung fibroblasts *in vitro* [12]. In this section, we tested the hypothesis that fumagillin, via MetAP2, would inhibit the proliferation of RPASMC *in vitro*. The effect of fumagillin on rat PASMC, a cell type that is actively proliferating in experimental PH [9], is unknown. We incubated primary RPASMC in serum-free medium, as described in Materials and Methods, to synchronize cells in G0. The cells were then released from growth arrest by addition of serum, and were incubated with or without PDGF, a pro-proliferative growth factor in smooth muscle cells [9], in the presence of fumagillin or vehicle control (Figure 9A). Cells were grown for one, two, and three days after the addition of serum. Cell number was determined by total SYBR Green intensity as measured by a fluorimetric plate reader. After the addition of serum, cell number increased in all conditions by day 1. By days 2 and 3, the increases in RPASMC number in the vehicle conditions were suppressed by fumagillin. Compared to RPASMC grown with the vehicle control, there were nearly 25% fewer cells incubated with fumagillin at days 2 and 3 after the addition of serum. Compared to RPASMC grown with the vehicle and PDGF, the addition of fumagillin decreased RPASMC number by nearly 33% at days 2 and 3 after the addition of serum. We also determined the rate of BrdU incorporation of RPASMC grown in the presence of fumagillin or vehicle control (Figure 9B–C). Cells grown in the presence of serum and fumagillin exhibited less BrdU incorporation than cells grown in the presence of serum and the vehicle. The apparent IC50 for the growth inhibition of fumagillin of RPASMC grown in the presence of serum is in the range of 2 nM, which is similar to published data in different cell types [12], [33]. Cells grown in the presence of serum, PDGF, and fumagillin also exhibited decreased BrdU incorporation compared to the vehicle control. Thus, fumagillin directly inhibits proliferation of RPASMC *in vitro*.

To determine if MetAP2 expression is necessary for proliferation, we incubated RPASMC in the presence of MetAP2-targeting short-interfering RNA (siRNA). Immunoblotting revealed suppression of MetAP2 expression in the absence of fumagillin (Figure 9D). In the presence of non-targeting RNA oligonucleotides and the vehicle, there was no inhibition of proliferation as measured by BrdU incorporation (Figure 9E). When incubated with MetAP2-targeting siRNA, RPASMC proliferation was inhibited. Incubation of fumagillin-treated cells where MetAP2 expression was suppressed by siRNA did not lead to any additional growth inhibition. These data support the hypothesis that MetAP2 expression is necessary for proliferation in RPASMC, and that the anti-proliferative effect of fumagillin is specific for MetAP2.

### PDGF Increases MetAP2 in Rat PA Smooth Muscle Cells

**Figure 10:**
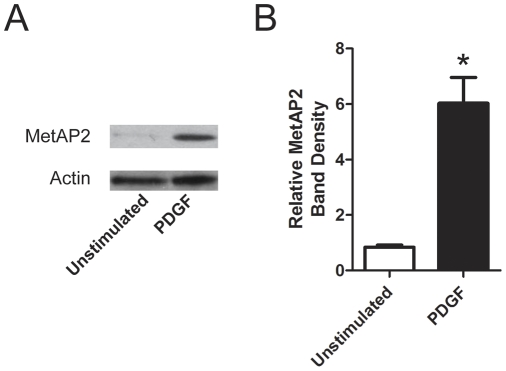
PDGF Increases MetAP2 in Rat Pulmonary Artery Smooth Muscle Cells. RPASMC were incubated in basal medium with 0.5% FBS. Cells were then stimulated with PDGF (10 ng/mL) for 18 hours. Cells were lysed in detergent and subjected to SDS-PAGE and immunoblotting for MetAP2 and Actin. (A) A representative immunoblot showing an increase in MetAP2 expression by PDGF. (B) Quantitative densitometry was performed as described in Materials and Methods and expressed as fold-increase compared to cells incubated in the absence of PDGF (mean + SEM, n = 3,*P<0.03). Data were analyzed by paired t-test.

Several growth factors that induce proliferation of smooth muscle cells and fibroblasts have been implicated in the pathogenesis of PH. One such growth factor is platelet-derived growth factor (PDGF) [9], [34]. We previously found that PDGF induced expression of MetAP2 in primary rat lung fibroblasts *in vitro* [12]. The effect of PDGF on MetAP2 expression in RPASMC is unknown. We hypothesized that MetAP2 expression would be increased downstream of PDGF. To test this, we used cultured RPASMC as a model system for thickening of vascular media in PH. RPASMC were cultured in the absence of serum with or without PDGF (Figure 10). Immunoblotting for MetAP2 revealed a nearly six-fold increase in MetAP2 protein expression in response to PDGF. Thus, PDGF, a pro-proliferative growth factor that is critical for the development of PH in animals [9], [35] and possibly in humans [9], [36] increased MetAP2 protein expression in RPASMC.

### The Effect of Fumagillin on the Proliferation and Death of Cells in the Pulmonary Arteries in MCT-Injured Rats

**Figure 11:**
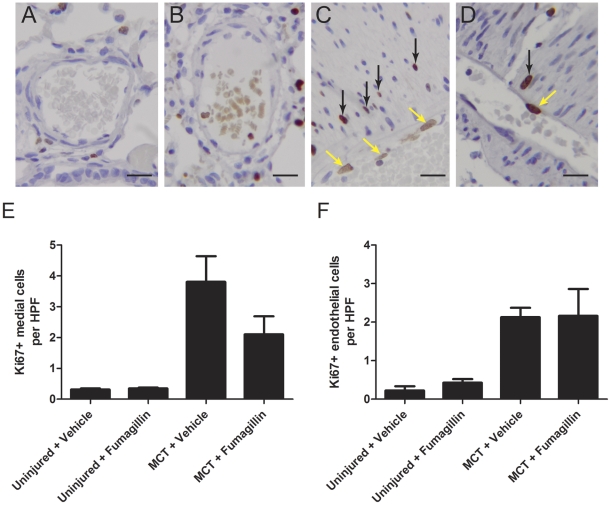
Quantification of Ki67 Staining in Rat Pulmonary Arteries. Immunohistochemistry for Ki67 was performed on rat lung sections from animals injured with MCT treated with or without fumagillin. Images of pulmonary arteries are shown (A) uninjured + vehicle (B) uninjured + fumagillin (C) MCT + vehicle and (D) MCT + fumagillin (Magnification ×400, bar = 20 µm). Black arrows point to Ki67+ nuclei in the medial layer of pulmonary arteries and yellow arrows point to Ki67+ nuclei in endothelial cells. Magnification ×400, inset line 20 mm. (E) Ki67+ nuclei in the medial layer (n = 4–6 per group) and (F) Ki67+ nuclei in endothelial cells of the pulmonary arteries (n = 4 per group) were quantified per hpf as described in Materials and Methods. MCT injury resulted in a nearly 15-fold increase in the number of Ki67+ nuclei in medial cells per vessel per hpf in the MCT-injured vehicle-treated lung. The increase in the number of Ki67+ nuclei was decreased nearly 30% with fumagillin treatment, but this difference did not reach statistical significance. Similarly, MCT significantly increased the number of endothelial cells staining positively for Ki67 nearly tenfold. However, no differences in the number of Ki67+ nuclei were detected after fumagillin treatment.

Because we observed that fumagillin decreased proliferation of RPASMC *in vitro*, we hypothesized that fumagillin treatment might specifically decrease proliferation of cells in the medial layer of pulmonary arteries in MCT-injured rats directly. And because fumagillin has well-known effects on endothelial cell proliferation [14], we also quantified the number of Ki67+ endothelial cells in the pulmonary arteries. We stained for the proliferation marker Ki67 [37] in lung sections from MCT-injured animals treated with fumagillin or vehicle at 28 days after MCT injury (Figure 11). Double staining for the smooth muscle marker, α-SMA, the endothelial cell marker, vWF, and Ki67 are shown in Figure S2. By visual inspection, abundant staining for Ki67 was present in the pulmonary arteries from MCT-injured, vehicle-treated animals compared to the pulmonary arteries of MCT-injured, early fumagillin-treated rats. Fewer Ki67+ cells were quantified in the animals treated with fumagillin three days after MCT injury compared to the vehicle controls, but this difference did not reach statistical significance. We also quantified Ki67+ cells in the medial layer of the pulmonary arteries in injured animals at two weeks following injury and found a similar trend to decreased Ki67 positivity in fumagillin-treated animals (*data not shown*). Furthermore, we found no significant differences in the number of Ki67+ endothelial cells in MCT-injured animals treated either with fumagillin or the vehicle. Thus, at four weeks after injury, there is no significant difference in the number of Ki67+ proliferating cells in the medial layer of the pulmonary arteries from MCT-injured rats treated with fumagillin compared to the vehicle control. We also performed terminal deoxynucleotidyl transferase dUTP nick end labeling (TUNEL) in parallel and found no significant differences in the number of TUNEL+ cells in animals that were treated with fumagillin or the vehicle (*data not shown*).

### MetAP2 Expression in Human and Rat PH

**Figure 12:**
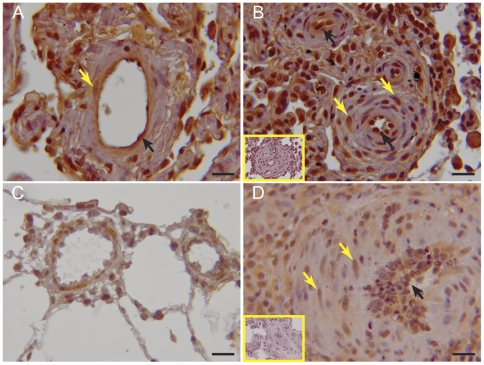
Detection of MetAP2 in human and rat lungs. Immunohistochemistry for MetAP2 was performed on formalin-fixed paraffin-embedded samples. Sections were counterstained with hematoxylin. Representative sections are shown, n = 3: (A) normal human lung (B) Pulmonary arterial hypertension (*inset* non-immune rabbit IgG) (C) normal rat lung (D) MCT-injured rat lung (*inset* non-immune rabbit IgG). Magnification ×400, bar = 20 µm. Note staining for MetAP2 in endothelial cells (black arrows) and cells in the medial layer (yellow arrows).

Having shown efficacy of fumagillin, a MetAP2 inhibitor, in a rat model of PH, we sought to determine if MetAP2, the target of fumagillin, is expressed in human disease. We performed immunohistochemistry for MetAP2 on multiple samples of lungs from patients with IPAH and from MCT-injured rats (Figure 12). MetAP2 staining was present in multiple cell types including endothelial cells and cells in the medial layer of the pulmonary vasculature. Very mild staining was present in the non-immune control. Double staining for the smooth muscle marker, α-SMA, the endothelial cell marker, vWF, and MetAP2 are shown in Figure S3. These results support the hypothesis that the cells in the pathologic lesions of human pulmonary arterial hypertension express MetAP2.

To determine if MetAP2 gene expression is increased in the lungs of patients with pulmonary arterial hypertension, we performed quantitative RT-PCR on lung samples from patients with IPAH (n = 6) compared to normal controls (n = 6). No significant difference in MetAP2 gene expression was detected between IPAH and normal controls (*data not shown*).

## Discussion

In this study, we tested the hypothesis was that fumagillin would prevent MCT-induced pulmonary hypertension by a direct anti-proliferative effect on rat pulmonary artery smooth muscle cells. We did indeed find that early treatment with fumagillin did prevent MCT-induced PH, but we discovered that the protective mechanism of fumagillin in MCT injury is far more complex than a simple effect on smooth muscle cell proliferation. We found that animals treated early with fumagillin did exhibit decreased vessel thickness. While fumagillin, and knockdown of its pharmacologic target MetAP2, did inhibit proliferation *in vitro*, we did not observe significant differences in the number of Ki67+ smooth muscle cells *in vivo*. We did uncover an anti-inflammatory effect of fumagillin following MCT injury, as we found fewer CD45+ leukocytes in the lungs of MCT-injured, fumagillin-treated animals compared to controls. But perhaps our most surprising observation was that late treatment of MCT-injured animals with fumagillin disconnected RV remodeling from PH.

The MCT-injured, late fumagillin-treated animals exhibited both decreased RV mass and decreased right ventricular cardiac myocyte cross-sectional area compared to the MCT-injured vehicle controls. We did not detect any clear differences between the number of capillaries in MCT-injured animals treated with fumagillin late or the vehicle suggesting that the effect of fumagillin in the heart was not a result of altered neoangiogenesis. We did find significantly reduced apoptosis in the heart of MCT-injured animals treated with fumagillin compared with the vehicle controls. We suggest that the effect of fumagillin on the myocardium is at least partially protective. MCT-injured rats treated with fumagillin beginning 14 d after injury exhibited decreased weight loss compared to the vehicle controls. While we found that MCT-injured animals treated late with fumagillin exhibited decreased cardiomyocyte hypertrophy compared to the MCT-injured, vehicle-treated controls, we did not detect significant differences in RV ejection fraction, RV end-diastolic pressure, RV minimum dP/dt, and Ees/Ea between these groups. We suggest that the risk of type II error in these observations is high. The measurement of these hemodynamic parameters in mechanically-ventilated animals following thoracotomy proved to be challenging. Because the intra-operative mortality of this procedure was high, the number of MCT-injured and vehicle-treated animals with adequate hemodynamic data was low. Little is known about the effects of fumagillin on the myocardium. Previous data support the efficacy of the fumagillin analogue TNP-470 in combination with cyclosporine in preventing myocardial arteriosclerosis after heart transplant in rats [38]. Our data lend further support to a growing body of literature that suggests that RV failure in PH is not solely explained by RV pressure afterload [39], [40]. Pulmonary artery banding studies have shown that there is RV hypertrophy with sustained PH but is associated with preserved RV function [41], [42]. In this model, the effects of MetAP2 inhibition, or off-target effects, by fumagillin on neurohormonal or growth factor signaling known to be important in right ventricular remodeling in MCT injury remain unknown. Further study of fumagillin will be necessary to elucidate the molecular mechanisms that govern right ventricular hypertrophy in animal models of PH.

What about the effect of fumagillin on endothelial cell proliferation? We found no effect of fumagillin on the number of microvessels in the heart following MCT injury or on proliferation of large vessel endothelial cells. It should be noted that a recent study found that the fumagillin analogue, TNP-470, when delivered subcutaneously at 30 mg/kg in MCT-injured animals, beginning at 21 d after injury, prevented the protective effects of estrogen by inhibition of pulmonary neoangiogenesis [43]. In addition, TNP-470 suppressed the "protective" cardiomyocyte hypertrophy in aorta-constricted mice expressing active heat shock transcription factor 1 (HSF1), an effect that the authors attribute to decreased angiogenesis [44]. The discrepancies between the effects of fumagillin and TNP-470 in MCT injury may be an effect of the dose of fumagillin that we employed, the route of administration, or the timing of fumagillin treatment. Our data suggest that the protective effects of fumagillin in MCT injury are certainly complex and involve effects of fumagillin on other cell types including inflammatory cells, smooth muscle cells, and cardiomyocytes.

The finding that fumagillin decreased both MCT-induced PH (this study) and bleomycin-induced pulmonary fibrosis [12] suggests that fumagillin may be effective in treating so-called “secondary” PH, that is World Health Organization Class III, PH, which is associated with hypoxemic diseases such as IPF. Further studies will be necessary to determine if fumagillin is efficacious in attenuating both pulmonary fibrosis and PH in animal models of secondary PH.

Why use MCT injury as a model for PH? Many correctly note that MCT injury is not associated with the plexiform lesion, the histopathologic hallmark of idiopathic pulmonary arterial hypertension in humans [3]. Moreover multiple agents have been shown to prevent MCT-induced PH, including agents that are associated with PH in humans [3]. Many cases of human PH are advanced at the time of diagnosis. Anti-proliferative approaches, such as fumagillin, for example, may have clinical utility, perhaps in conjunction with currently available therapies, in preventing disease progression. Perhaps most critical to developing any therapy for pulmonary hypertension is to determine the effect of the compound on the right ventricle. Death in PH is due to failure of the right ventricle. MCT injury, as opposed to chronic hypobaric hypoxia, is associated with significant PH, RV strain, and failure [3]. Further studies will be necessary to determine if fumagillin is effective in preventing cardiac remodeling in other animals of PH.

Our data show that MetAP2 expression is increased by PDGF in RPASMC *in vitro*. We suggest that MetAP2 exerts a permissive effect on RPASMC proliferation. But how does fumagillin inhibit proliferation of RPASMC? The enzymatic function of MetAP2 is to cleave the N-terminal methionine from nascent polypeptides during protein synthesis [15]. The removal of methionine is essential for further N-terminal modifications, for example acetylation by N-α-acetyltransferase and myristoylation of glycine by N-myristoyltransferase [16]. Growth inhibition induced by TNP-470 is dependent on p21 and p53 [33]. How the inhibition of the enzymatic function of MetAP2 leads to p21 induction is not clear [33]. Incorrect processing of a protein's N-terminus can theoretically result in aberrant biological functions and lead to a cell stress response [45]. Although the current study has implicated the enzymatic activity of MetAP2 in promoting proliferation, MetAP2 has other recognized non-enzymatic functions. It was first characterized in 1988 as a 67 kDa protein that binds to eukaryotic initiation factor-2α (eIF2-α), protecting it from phosphorylation and thus maintaining active protein synthesis [15]. Pharmacologic inhibition of MetAP2 by fumagillin-type compounds does not affect the ability of MetAP2 to protect eIF2-α from phosphorylation [46]. While our *in vitro* data support a specific effect of fumagillin for MetAP2, we also cannot exclude MetAP2-independent effects of fumagillin *in vivo* [47].

Fumagillin, and its analogues, have advanced into clinical trials in different malignancies [48], [49], [50]. Dose-limiting neurotoxicity has limited the application of fumagillin-like treatments in clinical practice. Typically these fumagillin doses reach ranges of 20–100 mg/kg. We chose intranasal delivery, at a significantly lower dosage (0.5 mg/kg). We found no efficacy of fumagillin delivered by intraperitoneal injection at 0.5 mg/kg (*data not shown*). Although use of the intranasal route for the delivery of fumagillin or its analogues has not been described in human clinical trials, the potential for delivery of high local concentrations of MetAP2 inhibitors might be expected to minimize neurotoxicity. Further studies will be required to determine the safety, effectiveness, and pharmacokinetics of inhaled fumagillin in human lung diseases.

## Supporting Information

Figure S1 **Movat staining of the pulmonary vasculature in MCT-injured rats.** Rats were injected with MCT and underwent treatment with fumagillin or vehicle control as described in Materials and Methods. Pulmonary artery thickness was measured by Movat staining as described in Materials and Methods. Representative images of vessels with similar luminal diameters are shown: (A) Uninjured + vehicle, (B) uninjured + fumagillin, (C) MCT + vehicle early, (D) MCT + fumagillin early, (E) MCT + vehicle late, and (F) MCT + fumagillin late (magnification ×400, bar = 20 µm).
(TIF)

Figure S2 **Double-label immunohistochemistry for Ki67, vWF, and α-SMA.** Double staining for Ki67 (brown) and the endothelial cell marker, von Willebrand Factor (gray). Yellow arrows point to a vWF+/Ki67+ cell. (B) Double staining for Ki67 (gray) and the smooth muscle cell marker, αSMA (brown). Yellow arrows point to an α-SMA+/Ki67+ cell. Inset image is the non-immune control for α-SMA (magnification ×400, bar = 20 µm).
(TIF)

Figure S3 **Immunofluorescent detection of MetAP2 and smooth muscle and endothelial cell markers.** Immunofluorescence for MetAP2 and α-smooth muscle actin (αSMA) and von Willebrand Factor (vWF) was performed on formalin-fixed paraffin-embedded samples. (A) MetAP2, (B) αSMA, (C) DAPI, and (D) merged image showing MetAP2 positivity in αSMA+ cells. (E) MetAP2, (F) vWF, (G) DAPI, and (H) merged image. White arrows point out MetAP2+ and vWF+ cells. Images A′–H′ show the corresponding non-immune controls (magnification ×400, bar = 50 µm).
(TIF)
