# Supplementary figures and images for: Early Treatment with Fumagillin, an Inhibitor of Methionine Aminopeptidase-2, Prevents Pulmonary Hypertension in Monocrotaline-Injured Rats
Source: PLoS One. 2012 Apr 11;7(4):e35388. doi: 10.1371/journal.pone.0035388 (PMC3324555; doi:10.1371/journal.pone.0035388)

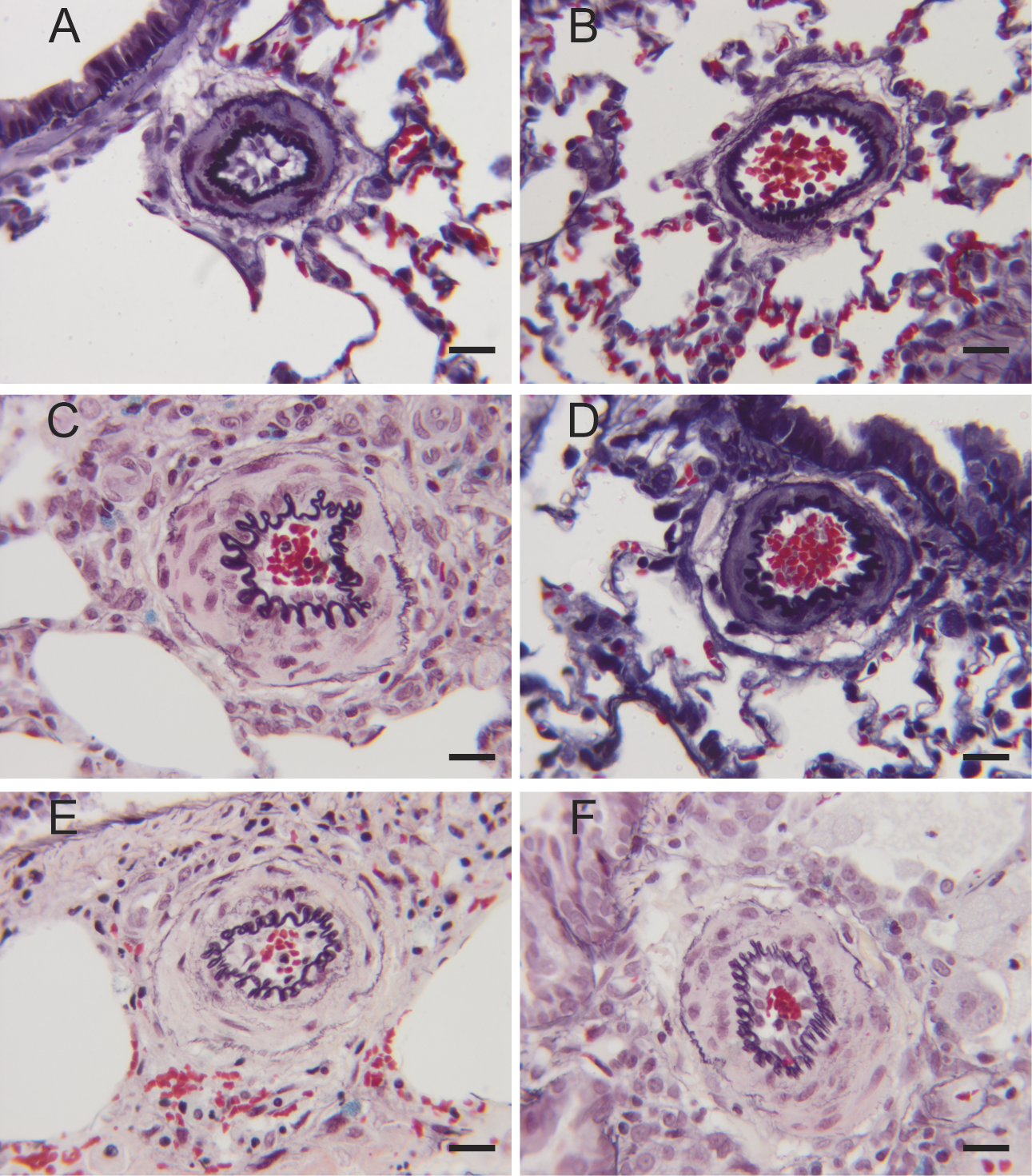

Supplement: Figure S1 — Movat staining of the pulmonary vasculature in MCT-injured rats. Rats were injected with MCT and underwent treatment with fumagillin or vehicle control as described in Materials and Methods. Pulmonary artery thickness was measured by Movat staining as described in Materials and Methods. Representative images of vessels with similar luminal diameters are shown: (A) Uninjured + vehicle, (B) uninjured + fumagillin, (C) MCT + vehicle early, (D) MCT + fumagillin early, (E) MCT + vehicle late, and (F) MCT + fumagillin late (magnification ×400, bar = 20 µm). (TIF) [file pone.0035388.s001.tif]

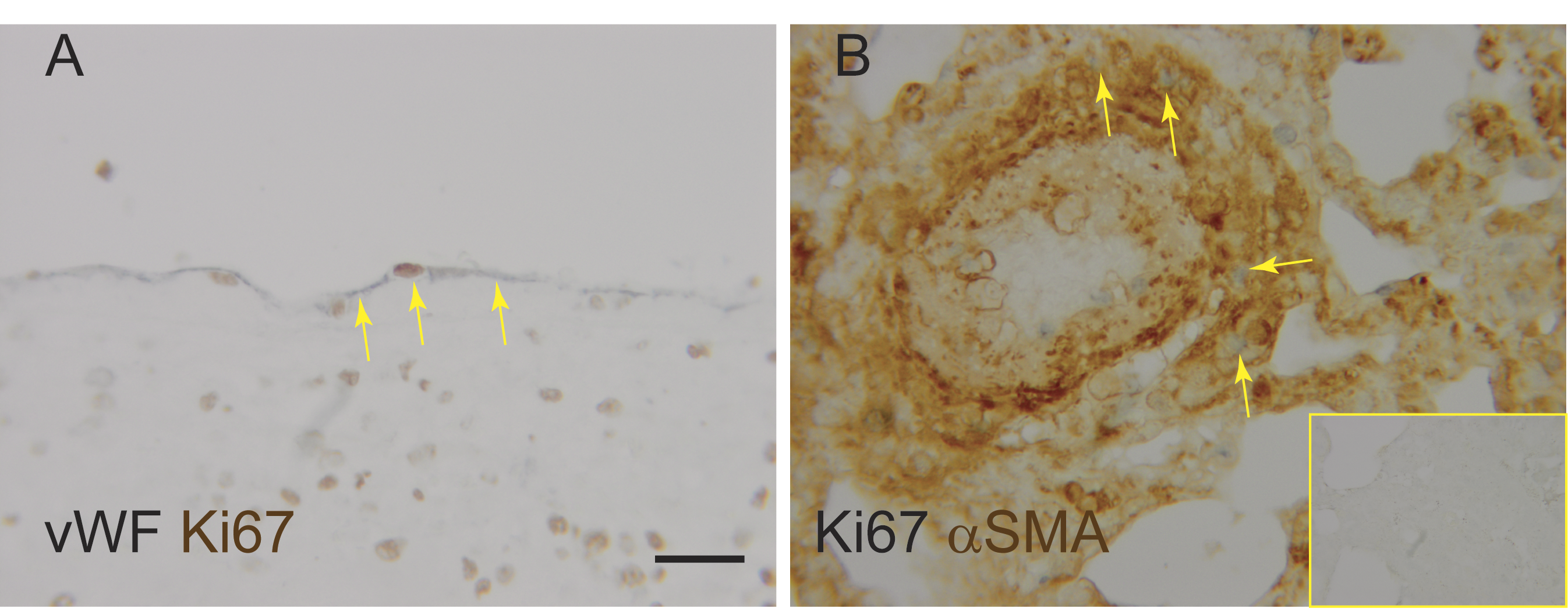

Supplement: Figure S2 — Double-label immunohistochemistry for Ki67, vWF, and α-SMA. Double staining for Ki67 (brown) and the endothelial cell marker, von Willebrand Factor (gray). Yellow arrows point to a vWF+/Ki67+ cell. (B) Double staining for Ki67 (gray) and the smooth muscle cell marker, αSMA (brown). Yellow arrows point to an α-SMA+/Ki67+ cell. Inset image is the non-immune control for α-SMA (magnification ×400, bar = 20 µm). (TIF) [file pone.0035388.s002.tif]

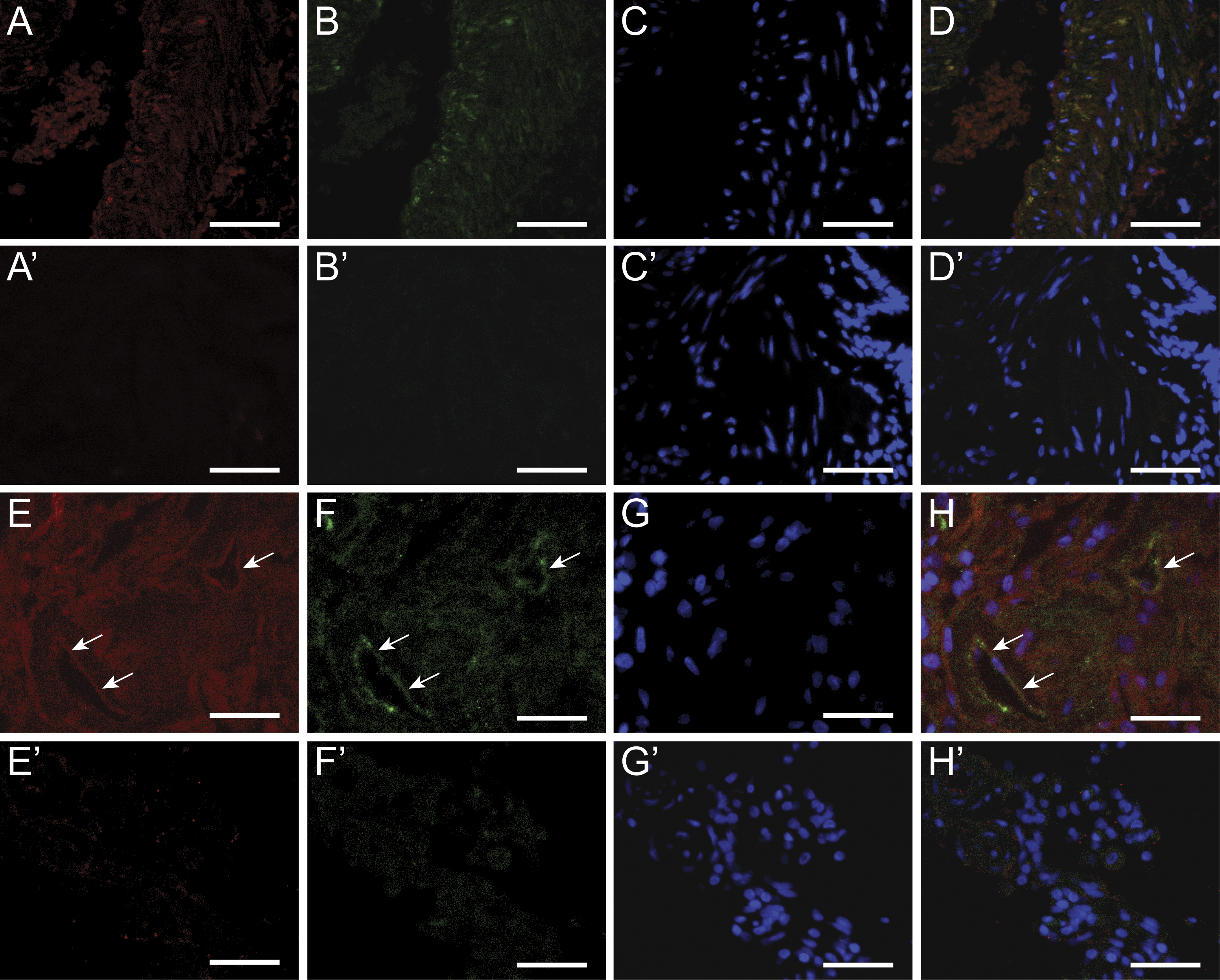

Supplement: Figure S3 — Immunofluorescent detection of MetAP2 and smooth muscle and endothelial cell markers. Immunofluorescence for MetAP2 and α-smooth muscle actin (αSMA) and von Willebrand Factor (vWF) was performed on formalin-fixed paraffin-embedded samples. (A) MetAP2, (B) αSMA, (C) DAPI, and (D) merged image showing MetAP2 positivity in αSMA+ cells. (E) MetAP2, (F) vWF, (G) DAPI, and (H) merged image. White arrows point out MetAP2+ and vWF+ cells. Images A′–H′ show the corresponding non-immune controls (magnification ×400, bar = 50 µm). (TIF) [file pone.0035388.s003.tif]
